# Supplementary material for: Correlative Study of Enhanced Excitonic Emission in ZnO Coated with Al Nanoparticles using Electron and Laser Excitation
Source: Sci Rep. 2020 Feb 13;10:2553. doi: 10.1038/s41598-020-59326-3 (PMC7018697; doi:10.1038/s41598-020-59326-3)
Supplement: Supplementary file 1 — Supplementary Information. [file 41598_2020_59326_MOESM1_ESM.docx]

Correlative Study of Enhanced Excitonic Emission in ZnO Coated with Al Nanoparticles using Electron and Laser Excitation.

Saskia Fiedler, Laurent O. Lee Cheong Lem, Cuong Ton-That, Markus Schleuning, Axel Hoffmann, Matthew R. Phillips

SUPPORTING INFORMATION.


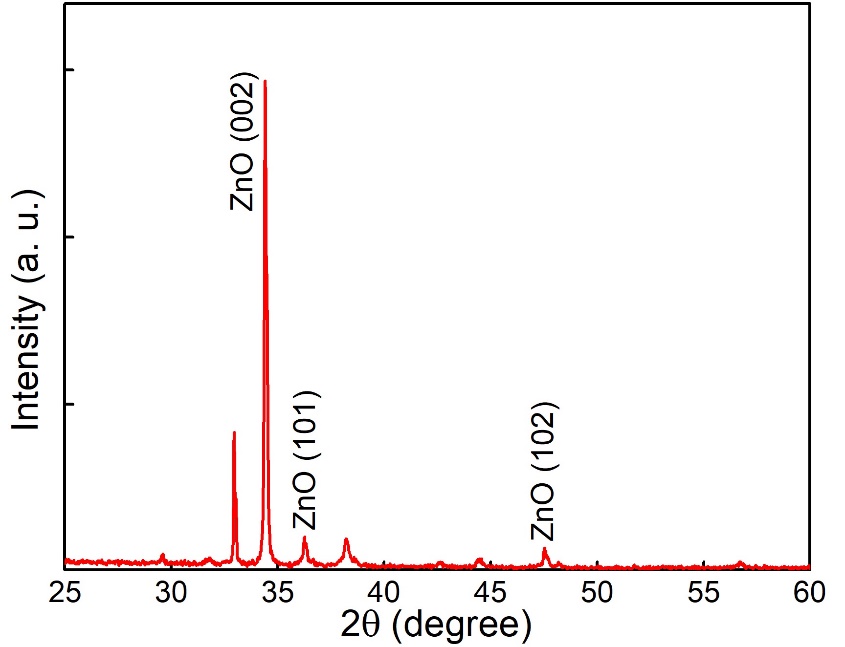


Figure SI 1. XRD spectrum of VS-grown ZnO nanorods on a Si substrate with ZnO (002) peak being most dominant indicating growth along *c*-axis.


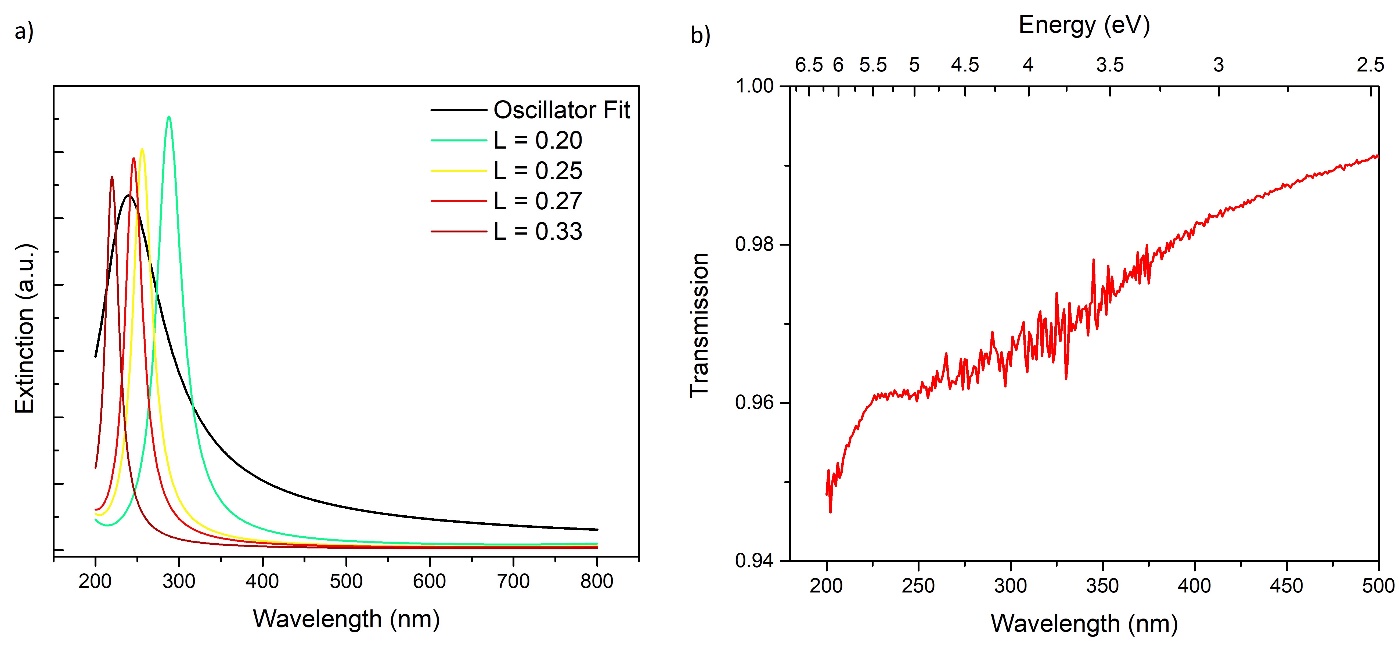


Figure SI 2. (a) Extinction spectrum of a 2nm thin Al film on a Si substrate fitted with an oscillator model, obtained by ellipsometry (black). Modelled extinction spectra of differently shaped Al nanoparticles in an Al_2_O_3_ matrix (colored lines), where L = 0.33 corresponds to a spherical nanoparticle and L = 0.20 a nanorod. The other L-values correspond to a more elliptical shape, being between those two L-values, indicating that the sample Al hot contains a large distribution of differently shaped Al nanoparticles. The ellipsometry results reveal that ~ 3% of the surface coating contains metallic Al NPs of different shapes and sizes that are covered or embedded in an Al_2_O_3_ matrix.

(b) Optical transmission spectrum of Al surface coating on UV-quartz divided by the reference spectrum of uncoated UV-quartz, showing characteristic Al NP LSPR absorption in the UV region of the spectrum.

The results above in (a) and (b) as well of the depth-resolved CL spectra in Fig 2, strongly suggest that the metal Al NPs are located at the ZnO surface covered by an Al_2_O_3_ matrix.
